# Efficiency clustering for low-density microarrays and its application to QPCR

**DOI:** 10.1186/1471-2105-11-386

**Published:** 2010-07-20

**Authors:** Eric F Lock, Ryan Ziemiecke, JS Marron, Dirk P Dittmer

**Affiliations:** 1 Department of Statistics and Operations Research, University of North Carolina, Chapel Hill, NC, USA; 2 Department of Microbiology and Immunology, Lineberger Comprehensive Cancer Center, University of North Carolina, Chapel Hill, NC, USA

## Abstract

**Background:**

Pathway-targeted or low-density arrays are used more and more frequently in biomedical research, particularly those arrays that are based on quantitative real-time PCR. Typical QPCR arrays contain 96-1024 primer pairs or probes, and they bring with it the promise of being able to reliably measure differences in target levels without the need to establish absolute standard curves for each and every target. To achieve reliable quantification all primer pairs or array probes must perform with the same efficiency.

****Results**:**

Our results indicate that QPCR primer-pairs differ significantly both in reliability and efficiency. They can only be used in an array format if the raw data (so called CT values for real-time QPCR) are transformed to take these differences into account. We developed a novel method to obtain efficiency-adjusted CT values. We introduce transformed confidence intervals as a novel measure to identify unreliable primers. We introduce a robust clustering algorithm to combine efficiencies of groups of probes, and our results indicate that using *n *< 10 cluster-based mean efficiencies is comparable to using individually determined efficiency adjustments for each primer pair (*N *= 96-1024).

****Conclusions**:**

Careful estimation of primer efficiency is necessary to avoid significant measurement inaccuracies. Transformed confidence intervals are a novel method to assess and interprete the reliability of an efficiency estimate in a high throughput format. Efficiency clustering as developed here serves as a compromise between the imprecision in assuming uniform efficiency, and the computational complexity and danger of over-fitting when using individually determined efficiencies.

## Background

Array and microarray based methods are the mainstay of molecular biology. Recently, lower-density or targeted arrays have been introduced. These comprise on the order of 10-1000 targets and represent an intermediate between 1 target assays, e.g. the viral load assay for HIV, and genomewide microarrays, comprising 10,000 -1,000,000 targets, e.g. Affymetrix™ arrays. Analytically they can be treated as either a collection of individual assays or as microarrays.

We are interested in discovering novel statistical approaches to the analysis of these intermediate density arrays. On the one hand high density microarray-based analysis methods do not capture all the information that is available. This results in lower than possible linear range and lower discriminating power. On the other hand, applying analysis methods developed for a single assay to arrays is overly complex and likely to introduce inacurracies due to overfitting problems.

Practically lower density arrays are based on real-time quantitative polymerase chain reaction (QPCR). Real-time QPCR measures the amount of product at each cycle of the reaction either by binding of a uorescent, double strand-specific dye (SYBR™ green) or by hybridization to a third sequence-specific, dual-labeled uorogenic oligonucleotide probe (molecular Beacon, TaqMan™ ). These have been used very successfully to profile messenger RNAs and microRNA [1-5]. Other assay formats use capture oligonucleotides or other means (e.g. Nanostring™ or Luminex™).

The simplest way to compare relative expression ratios is the so-called  method, which assumes (i) uniform and (ii) perfect efficiency: the amount of product doubles exactly after each PCR cycle for each primer in the array. However, most QPCR reactions do not attain optimal efficiency [6,7]. Even small deviations from an assumed efficiency level can lead to multiple-fold inaccuracies in expression measurements [8]. Therefore, differing efficiencies among primer-pairs, as well as the reliability of the efficiency estimates must be considered.

The most accurate and also most complex method is to calculate individual primer pair efficiencies or to use an absolute standard curve. This method does not assume uniform efficiency among different primer pairs and it does not assume perfect efficiency. The well known REST method [9] is an example of this strategy. However, this approach becomes impractical and extremely costly to use even for low density arrays.

More important, using individually adjusted efficiencies brings about the problem of overfitting and it requires that the individual efficiencies were determined with equal confidence. For instance, a 96 primer targeted QPCR array, would require the computation of 96^2 ^individual efficiency ratios. These need to be tracked throughout the analysis process and each ratio is associated with its own error, which also needs to be propagated throughout the analysis process.

The methods presented here use the serial dilution method [10] to obtain initial primer pair efficiencies. We expand on this method, introducing transformed normality-based confidence intervals as a novel tool to interpret the reliability of an efficiency estimate. We propose a rule to identify unreliable primers, as well as a robust algorithm to cluster primers with similar efficiencies. Finally, we show how differences in efficiency can be applied for more accurate comparisons of relative gene expression.

## Methods

### Experimental Methods

The primer arrays and experimental methods were previously described [11,12]. Primers were from commercial sources (MWG Inc.) and resuspended at 100 pmol per microliter in 0.1x Tris-EDTA ph 8.0. The QPCR reaction contained 2.5 microliter of primer mix at a starting concentration of 300 nM combined with 7.5 microliter SYBR Green 2× PCR mix (Applied Biosystems, Inc.) and 5 microliter target DNA. It was subjected to real-time QPCR on an Opticon2 cycler (MJR Inc.) using standard cycling conditions [13]. This particular QPCR array was directed against every open reading frame of human herpesvirus 8. This particular virus is made of linear double-stranded DNA. Thus we were able to use the same purified viral DNA as a common target for all primers.

### Quantitative Analysis

The R programming environment for statistical computing and graphics [14] was used for all computation and statistical analysis. Stand-alone R functions were developed to automate the analysis for a given serial dilution table. Functions were created to provide individual efficiency estimates with confidence, identify and remove unreliable primers, cluster amplification efficiencies, and adjust *C*_*T *_values in a table based on user-defined efficiencies. All functions were included in the R script Primer Efficiency Analysis (Additional file 1 - PEA.r); a reference manual is available with setup instructions, detailed function descriptions and illustrative examples (Additional file 2 - PEA User's Guide).

## Results and Discussion

### Estimating Efficiency with Confidence

**Figure 1:**
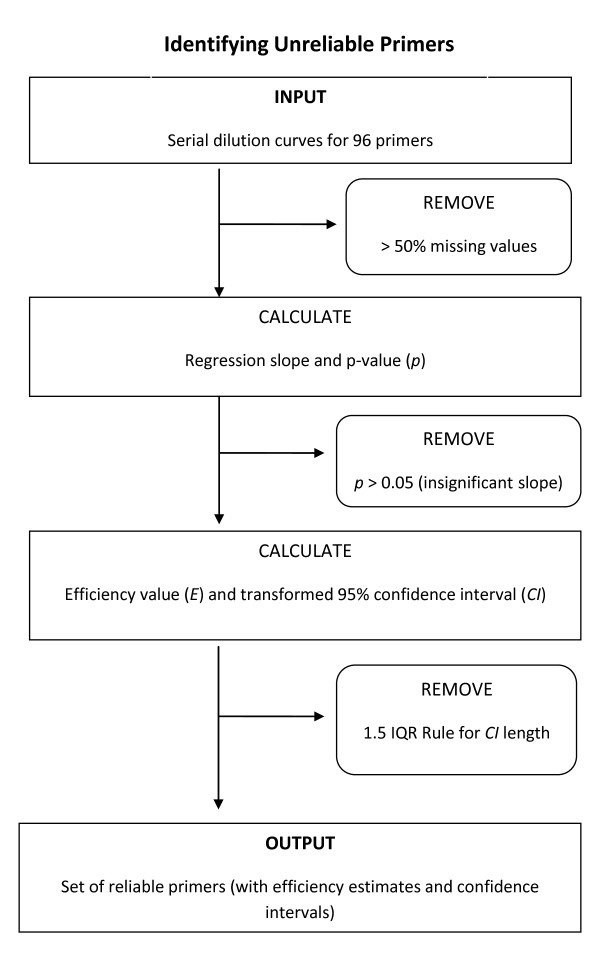
**Flow chart for estimating efficiency with confidence and identifying unreliable primers**.

We developed a novel algorithm for the adjustment of primer and probe efficiencies, specifically as it relates to low density real-time QPCR arrays. This approach is demonstrated using a dataset of 96 primers at four dilution levels. Figure 1 outlines the algorithm.

The number of cycles required to reach a certain level of product (*C_T_*) is measured for 96 primers, each with initial dilution levels 1, 0.1, 0.01, and 0.001. *C_T _*values above 40 indicate undetectable product and are considered to be missing values (NA) for subsequent calculations. If the dilution curve for a primer contains 2 or more missing values (50% of total), statistical analysis for a linear fit is impossible and the primer is removed from the analysis. Of the 96 original primers in our dataset, 3 were removed due to missing values.

**Figure 2:**
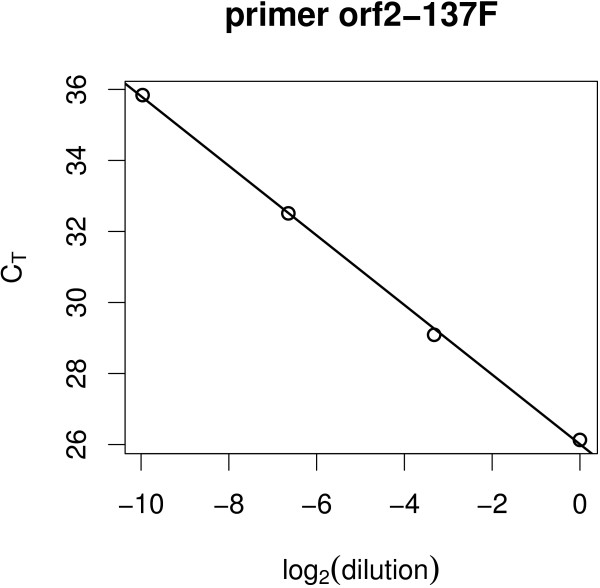
**Shown is an example of the experimental data that serve as input for further calculation**. The logarithm (*log*_2_) of the initial concentration is shown on the horizontal axis and the *C_T _*values on the vertical axis. *C_T _*values are measured at initial dilution levels 1, 0.1, 0.01 and 0.001 and exhibit a strong linear trend.

Amplification efficiency (*E*) estimates for each primer-pair are calculated using their serial dilution curve. Theoretically, one expects a linear relationship between *C_T _*and the logarithm of the initial dilution level. Therefore, we fit a standard linear model of the form(1)

for each primer, on the four levels of dilution. We use a base-2 logarithm (log_2_) rather than the common log_10 _[15], as the former has the nice interpretation that a unit slope (*β*_1 _= -1) corresponds to perfect efficiency (*E *= 2). Figure 2 shows the fitted linear model for a single primer-pair.

Before continuing, we remove those primers for which a linear fit is not statistically significant, based on the t-statistic for the estimated slope coefficient . If the corresponding p-value is above 0.05, the primer is considered unreliable and removed from further analysis. Using the t-statistic rather then the regression coefficient *R*^2 ^initially serves the following purpose. The t-statistic accounts for the number of dilution steps, whereas a threshhold based on the coefficient of determination *R*^2 ^does not (*R*^2 ^= 1 for 2 data points and likely decreases with the addition of more measurements). More dilution steps generally result in more accurate efficiency estimates. In our dataset, 8 primers were removed due to an insignificant linear slope.

Standard normality-based methods are then used to construct a confidence interval for the slope parameter *β*_1_. For each primer a 95% confidence interval for the slope, (*β_lower_*; *β_upper_*), is given by(2)

where  is the estimated slope, t_0.025;*n*-2 _is the 2.5% quantile of Student's t-distribution with *n *- 2 degrees of freedom, and SE() is the standard error of. This analysis used 3 or 4 dilution levels for each primer (*n *= 3 or 4), so we use a t-distribution with 1 or 2 degrees of freedom.

The estimated slope of the log-transformed regression model () is used to estimate the amplification efficiency (E) of each primer through the transformation(3)

As this transformation is strictly increasing for *β*_1 _< 0, confidence intervals for *β*_1 _are easily extended to confidence intervals for *E*. Under the condition *β_upper _*< 0, a 95% confidence interval for *E *is given by (*E_lower_*;*E_upper_*), where(4)

and(5)

Only keeping those primers with a negative and significant slope ensures that the condition *β_upper _*< 0 is satisfied.

### Identifying Unreliable Primers

Bartlett's test of heteroscadasticity (unequal variability within groups), applied to the residuals of each linear model fit by (1), is highly significant (*p *< 0.0001). This indicates that certain aberrations in efficiency estimation may be due to inherent primer reliability issues, rather than standard experimental and residual error. Purging those primers with missing values and an insignificant slope can be considered a first step to identify primer-pairs yielding unreliable data, but further analysis is warranted. Here we introduce transformed confidence intervals as a novel measure of primer reliability

The length of the transformed confidence interval *E_upper _*- *E_lower _*is used to quantify the precision of the estimated amplification efficiency . To identify those primers with unreliable data, we first calculate the interquartile range (IQR) of the transformed CI lengths as the difference betweeen the 75th and 25th percentiles (quantiles). Any primer with a transformed CI length higher than 1.5 × *IQR *above the 75th percentile is identified as unreliable. Such a procedure (the "1.5 × *IQR*" rule) is commonly used to identify outliers. In our dataset, 7 primers are identified using this procedure.

**Figure 3:**
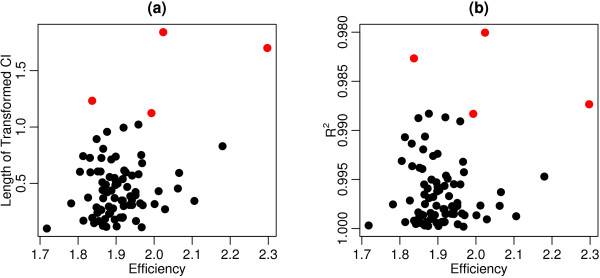
**In panel (a) length of the transformed confidence intervals are shown on the vertical and primer efficiency on the horizontal axis**. In panel (b) the squared regression coefficient is shown on the vertical and primer efficiency on the horizontal axis. Transformed CI outliers are colored red in both panels. Outliers not shown are primers 73-5'UTR, orf24-306F and orf35-200F, with transformed CI lengths 6.95, 58.0 and 4.93 × 10^15^, respectively.

Figure 3 shows the estimated amplification efficiency E, transformed CI length, and *R*^2 ^value of the original linear model for each primer. The coefficient of determination *R*^2 ^measures the strength of a linear fit, and can be interpreted as the percentage of variation in the response (*C_T_*) that is explained by *log*_2_(*Dilution*). Primers identified as outliers by the 1.5 × *IQR *rule for transformed CI length are colored red. After removing outliers based on CI length, the remaining primers all show strong linearity with *R*^2 ^values greater than 0.98 and correlation coefficients greater than 0.99.

Most of those primers identified for large transformed CI's tend to have lower *R*^2 ^values, whereas there is no correlation with primer pair efficiency. Hence, a low efficiency primer pair may indeed be very reliable and an apparently good efficiency primer may be the result of a bad fit. This demonstrates that considering a measure of variation for each efficiency estimate adds value over prior methods that consider efficiency as the sole criterion for primer performance.

**Figure 4:**
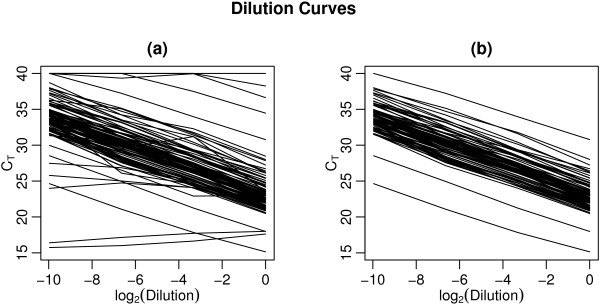
**Relationship between *C_T _*and log_2_(dilution) for all 96 primers before (a) and after (b) removing relatively unreliable primers**. *C_T _*values are shown on the vertical and log_2 _dilution of input target on the horizontal axis. Note the purged data is much "cleaner", with non-linear and sporadic relationships removed.

Before purging the data of unreliable primers, the mean estimated efficiency is 1.94, with standard deviaton 0.180; afterward the mean efficiency is 1.90 with standard deviation 0.071. This illustrates our contention that calculating a measure of the efficiency estimate improves overall performance vis-à-vis strategies that rely on total efficiency alone. For this particular array, the mean efficiency was lower after purging, but uniformity across all primers in the array improved resulting in a reduced standard deviation. Figure 4 shows the relationship between *C_T _*and log dilution level for every primer (4a), and after removing missing values, insignificant linear slopes and CI length outliers (4b). The purged data is much "cleaner", with non-linear and sporadic relationships removed. This purged set of primers is appropriate for use in subsequent, rank-based, clustering and classification analyses, whereas the original set would produce unacceptable results.

### Comparing Primer Efficiencies

Even after purging the data of unreliable primers, estimates of primer efficiency vary, from *E *= 1.7 to *E *> 2. It has been shown that rather than improving accuracy, individual corrections to efficiency can in fact exaggerate artificial differences in expression [16]. Therefore, we consider the hypothesis that there is no underlying difference in primer efficiencies, and observed differences are due to residual error.

After removing those primer-pairs with relatively unreliable data, Bartlett's test of heteroscadasticity is no longer significant at the 5% level. This indicates that differences in primer variability among the purged data may be negligable. The Shapiro-Wilk normality test fails to reject the hypothesis that the residuals for each fitted dilution curve (see Equation 1) come from the same univariate normal distribution (p = 0.3569). Hence the basic assumptions for an Analysis of Variance (ANOVA) are satisfied, and we consider the fitted dilution curves as a single multivariate model, with a different intercept and slope for each primer:(6)

The F-statistic for the interaction between primer and log dilution *log*_2_(*dil*) is highly significant (*p *= 0.0017). This indicates that differences among slope coefficients, hence the resulting efficiency estimates, are not merely due to residual error.

This significant difference in primer efficiencies shows that simply using the average efficiency (*E *= 1.90) among all primers is inappropriate for comparing expression levels. An alternative approach is to use the individually determined efficiency value for each primer. Both methods generate errors on efficiency, which may be propogated through the calculation of expression quantities [17].

A trade-off exists between the lack of precision in not recognizing differences in primer efficiency, and the computational complexity and danger of over-fitting when using individually determined efficiencies for each primer. Here we introduce a novel, third alternative, which finds a useful balance between the two extreme approaches. We first cluster primer-pairs based on their estimated efficiency values, then use only a measure of the average efficiency within each cluster for subsequent adjustments. Rather than a single efficiency, i.e. no correction, we use 5-10 different efficiencies, based on clustering to obtain adjusted *C_T _*values. Rather than 96-1024 individual, but computational unreliable, efficiencies, we use 5-10 different cluster-derived efficiencies, knowing that within each cluster, there exists no significant difference between individual primer pairs.

We consider a simple efficiency clustering algorithm based on the fitted ANOVA model (5), yet with only one slope coefficient for each "group":(7)

**Figure 5:**
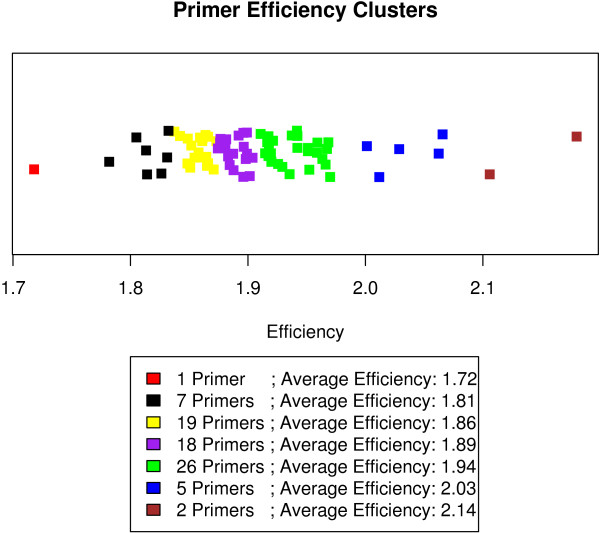
**Plot of individually determined primer efficiency values**. For the 78 primer pairs considered individual efficiency estimates range from 1.72 to 2.2, and these primer pairs are grouped into 7 clusters. These clusters are color coded. Shown below is a table of the numbers of primers in each cluster and the average efficiency. Within-cluster differences in efficiency are insignificant, and the average efficiency for each cluster is used.

First two groups of primers, corresponding to two dilution slopes, are selected in a way that maximizes the power of the model (as measured by *R*^2^). If the model with two groups significantly improves over the model with a single slope (the p-value of the corresponding F-statistic is less than 0.05), we divide the two groups into three optimal groups of primers. If three groups represents a significant improvement over two, we divide the primers into four groups, and so on. For this particular dataset, we find that the optimal model with eight slope coefficients does not significantly improve on the model with seven, hence we identify seven clusters. The primer clusters and corresponding efficiencies are shown in Figure 5.

Note that the majority of efficiencies, even across clusters, still are between E = 1.8 and E = 2.0. This is expected since we started with a "good" primerset based on sequence criteria [11]. Still we are able to subdivide the "efficiency space" 1.8 to 2.0 into four clusters and thus improve overall performance. Furthermore, we are able to use even the relatively divergent primers pairs with *E *< 1.8 and *E *> 2 (due to repeats in the target sequence) for further analysis thus maximizing usability. The clustering method described is robust in the sense that significant differences in primer efficiencies should be reflected in the number of clusters. Both the magnitude of observed efficiency differences and precision of those efficiency estimates are accounted for. Arrays where the observed differences in primer efficiency are not significant are expected to result in few clusters, whereas if efficiencies differ greatly and can be measured precisely the method will tend to identify many clusters.

### Comparison of Methods

Does the use of mean cluster efficiencies perform better than alternative approaches for the comparative analysis of relative RNA expression levels? To answer this question, we proceeded as follows. Define the adjusted fold change (AFC) for a reaction as the estimated fold change (*E^CT^*) divided by the initial dilution:(8)

For replications of the same experiment with different initial dilutions, we expect the AFC to be the same. However, an inaccurate assumed efficiency *E *will lead to different AFC values. For each primer, we calculate the coefficient of variation for the AFC values corresponding to the four dilution levels. The coefficient of variation (CoV) is defined as the standard deviation of the four values, divided by their mean.

**Figure 6:**
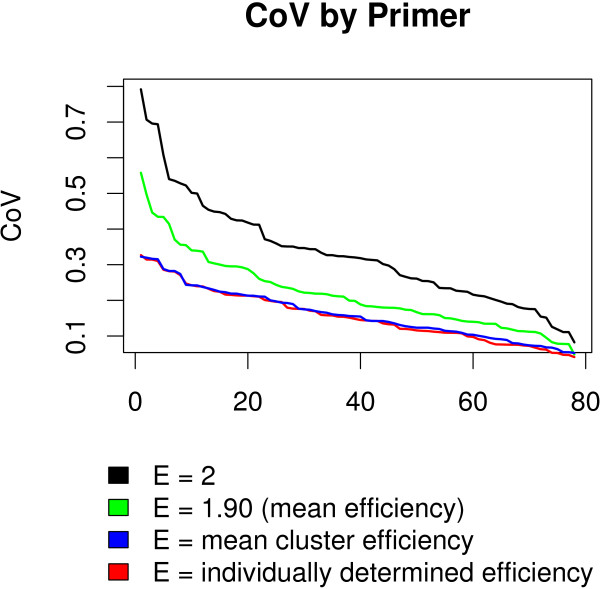
**Plot of Coefficient of variation (CoV) of AFC values for four efficiency estimation methods**. For each method, primer CoV values for each of the 78 primers considered are sorted from largest to smallest. A precise efficiency estimate results in a lower CoV. The curves for clustered and individually determined efficiencies are overlapping. This demonstrates that using just a small number of average cluster efficiencies is comparable to using individual primer efficiencies, i.e. the differences in efficiency within each cluster are negligible.

Similar AFC values among different dilutions result in a CoV close to 0; dissimilar values result in a CoV closer to 1. The CoV values for each primer, under different assumed efficiencies, are shown in Figure 6. Using a single universal efficiency *E *= 2 yields the worst result, i.e. the largest CoV across all primers (Figure 6, black line). Using instead the experimentally determined single mean efficiency (*E *= 1.90) across the array improves the CoV significantly (Figure 6, green line). Using the 7 average efficiencies by cluster lowers the CoV even further overall (Figure 6, blue line). As expected it improves most dramatically the performance of the most divergent primers, i.e. the tails (primer 1-10 and 70-80). Notably, the curves for the 7 average efficiencies by cluster and for the individually determined efficiencies (Figure 6, red line) are overlapping. This demonstrates that using just a small number of average cluster efficiencies is as good as using individual primer efficiencies, i.e. the differences in efficiency within each cluster are negligible.

### Comparing Relative Expressions

The improvements based on mean cluster efficiencies are easily incorporated into existing analysis workflows, by computing adjusted *C_T _*, which we call . These are defined as follows. The relative expression ratio between a reaction using primer pair A and a second reaction using primer pair B is given by(9)

where *E_A _*and *E_B _*are the efficiencies of primer pair A and B, respectively. If the two primers are assumed to have the same efficiency *E *(e.g. they belong to the same efficiency cluster), the calculation is simplified:(10)

This insight is captured for group-wise comparison in the REST [9] software package, which also incorporates error estimates and tests of significance, while allowing for manual input of PCR efficiencies.

The alternative proposed here, uses a direct comparison of *C_T _*values after adjusting for amplification efficiency. We propose the following adjustment:(11)

Here,  is the estimated number of cycles required under perfect efficiency, so that the true fold difference is given by . For example, if a reaction has perfect efficiency (*E *= 2) with observed *C_T _*= 24, while another has sub-perfect efficiency (*E*= 1.8) with observed *C_T _*= 28.5, we calculate  = 24 for both reactions, indicating no difference in expression. A practical advantage of using  is that the data format of the original set-up remains the same. The expression data remain log transformed and can be used directly in any microarray profiling software such as Eisen's original clustering program [18]. Calculating  is a preprocessing step that does not change the runtime or memory requirements of the subsequent analysis programs.

Table 1 gives raw and adjusted *C_T _*values for seven primer pairs with different efficiencies. Multiple-fold differences are observed between estimated expression under perfect efficiency and expression after correcting for imperfect efficiency. This suggests that the assumption of perfect efficiency can often lead to multiple fold inaccuracies in relative expression.

**Table 1:** Adjusted Relative Expression.

Adjusted Relative Expression				
**Primer**	**Cluster efficiency**	***C_T_***	**Adjusted *C_T _*: **	**Fold difference: **

orfK6	1.72	24.15	18.85	39.28
orf7-1632F	1.81	23.71	20.39	10.00
orf21-1578F	1.86	23.85	21.32	5.78
orf4-1511F	1.89	23.29	21.38	3.75
orf9-708F	1.94	24.24	23.16	2.12
orf2-137F	2.03	26.13	26.76	0.64
tac29-5F	2.14	25.78	28.34	0.17

Raw and adjusted *C_T _*values are shown for seven primer pairs, belonging to different efficiency clusters. Fold difference is calcuated as the estimated concentration under perfect efficiency, divided by concentration under the adjusted efficiency. The assumption of perfect efficiency can lead to multiple fold inaccuracies in relative expression.

## Conclusions

As array-based measurements for DNA, mRNA and microRNA levels migrate into the mainstay of molecular biology, failure to carefully consider the efficiency of each individual reaction or assay can lead to significant measurement inaccuracies. Yet, explicitly calculating and considering individual assay characteristics is not feasible even for low-density arrays. This is an important problem particularly for real-time QPCR based arrays, but it applies to any type of microarray. QPCR primers differ significantly both in reliability and amplification efficiency, hence they need to be experimentally validated. (i) We identified transformed confidence intervals as a useful means to assess and interpret the reliability of an efficiency estimate. Transformed confidence intervals provide a novel, independent measure in addition to calculation of primer efficacy E, with which to assess primer/probe quality. (ii) After purging unreliable estimates we propose a robust clustering algorithm to group efficiencies, reducing computational complexity and potential over-fitting. Our results suggest that use of a limited number of clustering-based efficiencies is comparable to use of individually determined efficiencies for each primer or probe.

## Authors' contributions

RZ performed the initial analysis of transformed confidence intervals. EL performed the clustering analysis, wrote the final R package and co-wrote the article. DD conceived the project and approach, and co-wrote the article. JM contributed expertise and ideas. All authors read and approved the final manuscript.

## Supplementary Material

Additional file 1 **PEA.r**. An R script to provide individual efficiency estimates with confidence, identify and remove unreliable primers, cluster amplification efficiencies, and adjust *C_T _*values; requires the R programming environment for statistical computing and graphics [14]. Click here for file

Additional file 2 **PEA User's Guid**. A reference manual for PEA.r; includes setup instructions, function descriptions and illustrative examples. Click here for file
