# Supplementary material for: Efficiency clustering for low-density microarrays and its application to QPCR
Source: BMC Bioinformatics. 2010 Jul 20;11:386. doi: 10.1186/1471-2105-11-386 (PMC2912893; doi:10.1186/1471-2105-11-386)
Supplement: Additional file 2 — PEA User's Guid. A reference manual for PEA.r; includes setup instructions, function descriptions and illustrative examples. [file 1471-2105-11-386-S2.PDF]

R package ‘Primer Efficiency Analysis’ (PEA.r)

This is a reference manual for a collection of R functions used to estimate primer efficiency values with confidence intervals, identify unreliable primer estimates, and cluster efficiency values. All functions are included in the R script ‘PEA.r’. For questions, contact lock@email.unc.edu.

Table of Contents

Loading the package into R…………..2
Input data…………………………………….2
Functions
    Identifying unreliable primers: *IdentifyUnreliable()*……………..3
    Clustering primer efficiencies: *ClusterEffs()*………..……………..….4
    Estimating efficiency with confidence: *AdjustCt()*……………...5

Loading the package into R

After you have saved the script 'PEA.r' somewhere on your computer's hard drive, you can load it into the R system by using the command *source()*. For more information on the *source()* command, enter *help(source).* After the script has been loaded, the functions contained in 'PEA.r' can be used normally.

EXAMPLES

If 'PEA.r' was saved in the folder *C:\R_Functions\,* load it into R with the command *source("C:\\R_Functions\\PEA.r")* (Use '\\' instead of a single '\').

If 'PEA.r' is saved in your working directory, you don't need to enter the entire file path: *source("PEA.r")* should be enough.

Input Data

As input, the functions contained in 'PEA.r' take a table of Ct values measured for various initial dilution levels, on different primer-pairs. Each row gives a different primer. By default, the first two columns in a table are taken to be the well number and name for each primer, while the remaining columns represent different initial dilutions. For example, an array with initial dilution levels 1, 0.1, 0.01 and 0.001 may have the following form:

```
> Data
   Well       primer X1.000 X0.100 X0.010 X0.001
1    A1   orf2-137F  26.13  29.09  32.51  35.84
2    A2  orf4-1511F  23.29  27.02  30.57  34.05
3    A3  orf6-2093F  25.41  29.23  33.21  36.08
4    A4  orf7-1632F  23.71  27.40  31.20  35.73
5    A5  orf8-2305F  24.24  28.31  32.20  35.83
6    A6   orf9-708F  24.24  27.39  31.62  34.62
7    A7 orf10-1065F  22.29  26.24  30.04  32.99
8    A8  orf11-983F  21.08  24.08  25.02  25.79
9    A9  orf16-200F  22.14  25.77  29.33  33.17
10  A10   orf17-59F  21.23  24.58  27.10  31.52
11  A11  orf18-690F  22.28  25.74  29.20  32.47
12  A12  orf19-480F  23.23  27.27  30.67  33.45
13   B1  orf20-622F  18.00  17.72  17.15  16.40
14   B2 orf21-1578F  23.85  27.86  31.57  34.97
15   B3   orf22-14f  23.58  27.56  31.06  34.84
16   B4 orf22-1017F  23.04  26.99  30.57  34.20
```

Figure 1

To load the data into R from a text file, use the command *read.table()* if the values are separated by a space or *read.csv()* if the values are separated by a comma. To load data from an Excel spreadsheet, the easiest thing to do is first save the file in '.csv' format, then load the data into R through the *read.csv()* command. For more information on importing data into R, see http://stat.ethz.ch/R-manual/R-patched/doc/manual/R-data.html.

Functions

IdentifyUnreliable(Data, Dilution, Display = FALSE)

Description: Use *IdentifyUnreliable()* to identify primers with unreliable data and remove them from the analysis.

Input:

Data - A table of Ct values measured for various initial dilutions, on different primers (see Figure 1).

Dilution – A vector giving the initial dilution levels (e.g. *c(1, 0.1, 0.01, 0.001)*).

Display - If *TRUE*, gives a scatter plot of efficiency estimates vs. transformed confidence interval (CI) length for each primer, with outliers colored red (see Figure 2). Primers with a transformed CI length greater than 3 are not shown. Default value is *FALSE*.

Output:

Insignificant – Primers with a statistically insignificant linear slope.

Unreliable – Primers identified as unreliable by the 1.5*IQR rule for transformed CI length.

Purged – Table in the same form as the input data, but with unreliable primers removed.

Example:

```
> NewData <- IdentifyUnreliable(Data, Dilution = c(1, 0.1, 0.01, 0.001), Display = TRUE)
> NewData$Insignificant
[1] orf11-983F  orf20-622F  orf33-852F  orf53-149F  orf64-5841F 71primer2
[7] K5-37F      K5-212F     K6-191F     K15-23F
> NewData$Unreliable
[1] orf17-59F   orf24-306F  orf35-200F  orf59-481F  orf63-2072F 73-5'UTR
[7] k11-55F
> NewData$Purged
  Well      primer   X1.000 X0.100  X0.010  X0.001
1   A1   orf2-137F   26.13    29.09   32.51   35.84
2   A2  orf4-1511F  23.29    27.02   30.57   34.05
….
```

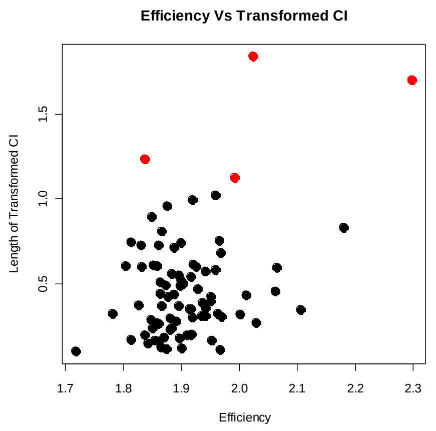

Figure 2

ClusterEffs(Data, Dilution, Display = FALSE)

Description: Use *ClusterEffs()* to cluster primers with similar efficiencies. It is recommended to first run *IdentifyUnreliable()* to purge the data of unreliable primers.

Input:
Data - A table of Ct values measured for various initial dilutions, on different primers (see Figure 1).

Dilution – A vector giving the initial dilution levels (e.g. *c(1, 0.1, 0.01, 0.001)*).

Display - If *TRUE*, gives a jitter plot of estimated primer efficiencies, colored by cluster(see Figure 3). Default value is *FALSE*.

Output:
Efficiencies – Efficiency estimates for each primer, using the average efficiency within a cluster. Efficiencies are given in a vector, in the same order as the input data.

Example:
```
> Efficiencies <- ClusterEffs(NewData$Purged, Dilution = c(1, 0.1, 0.01, 0.001), Display = TRUE)
Found 7 clusters.
> Efficiencies
 [1] 2.033951 1.889696 1.889696 1.814887 1.814887 1.939084 1.889696 1.889696
 [9] 1.939084 1.939084 1.858131 1.858131 1.858131 1.939084 1.858131 1.858131
  ….
```

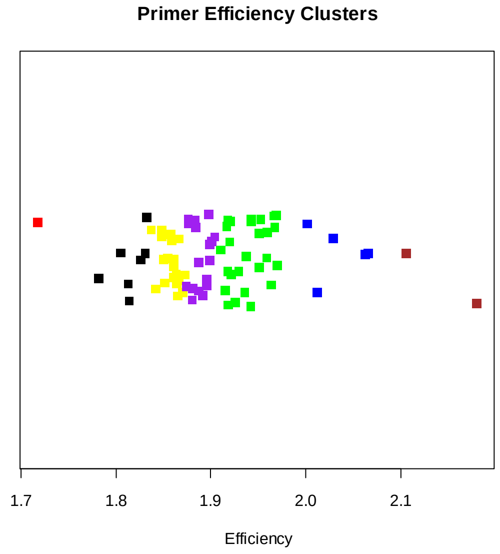

Figure 3

AdjustCt(Data, Efficiencies)

Description: Use *AdjustCt()* to adjust Ct values within each row by a given efficiency.

Input:
Data - A table of Ct values measured for various initial dilutions, on different primers (see Figure 1).

Efficiencies – Vector of efficiencies, corresponding to each row of the data.

Output:
Adjusted – Table in the same form as *Data*, but with adjusted Ct values.

Example:
To purge unreliable primers, cluster efficiencies, then adjust Ct based on the clustered efficiencies:

```
> NewData <- IdentifyUnreliable(Data, Dilution = c(1, 0.1, 0.01, 0.001))
> ClusEffs <- ClusterEffs(NewData$Purged, Dilution = c(1,0.1,0.01,0.001))
Found 7 clusters.
> Data_Adjusted <- AdjustCt(NewData$Purged, ClusEffs)
> Data_Adjusted
  Well     primer      X1.000      X0.100     X0.010     X0.001
1   A1   orf2-137F   26.76456   29.79645  33.29950  36.71037
2   A2  orf4-1511F   21.38380   24.80852  28.06796   31.26314
3   A3  orf6-2093F   23.33029   26.83764  30.49189   33.12699
4   A4  orf7-1632F   20.38774   23.56070   26.82824  30.72350
….
```
